# PRIMARY TUMOR LYMPHOVASCULAR INVASION NEGATIVELY AFFECTS SURVIVAL AFTER COLORECTAL LIVER METASTASIS RESECTION?

**DOI:** 10.1590/0102-672020210001e1578

**Published:** 2021-06-11

**Authors:** Renato Gomes CAMPANATI, João Bernardo SANCIO, Lucas Mauro de Andrade SUCENA, Marcelo Dias SANCHES, Vivian RESENDE

**Affiliations:** 1 Department of Surgery, Faculty of Medicine, Federal University of Minas Gerais, Belo Horizonte, MG, Brazil

**Keywords:** Colorectal neoplasms, Neoplasm metastasis, Prognosis, Survival analysis, Neoplasias colorretais, Metástase neoplásica, Prognóstico, Análise de sobrevida

## Abstract

***Background*:**

About 50% of the patients with colorectal adenocarcinoma will present with liver metastasis and 20% are synchronic. Liver resection is associated with improvement in survival in comparison to chemotherapy alone.

***Aim*:**

To analyze the overall survival in patients submitted to liver resection of colorectal cancer metastasis and prognostic factors related to the primary and secondary tumors.

***Methods*:**

A retrospective analysis of a prospectively maintained database regarding demographic, primary tumor and liver metastasis characteristics.

***Results*:**

There were 84 liver resections due to colorectal cancer metastasis in the period. The 5-year disease-free and overall survivals were 27.5% and 48.8% respectively. The statistically significant factors for survival were tumor grade (p=0.050), lymphovascular invasion (p=0.021), synchronous metastasis (p=0.020), as well as number (p=0.004), bilobar distribution (p=0.019) and diameter of the liver metastasis over 50 mm (p=0.027). Remained as independent negative predictive factors: lymphovascular invasion (HR=2.7; CI 95% 1.106-6.768; p=0.029), synchronous metastasis (HR=2.8; CI 95% 1.069-7.365; p=0.036) and four or more liver metastasis (HR=1.7; CI 95% 1.046-2.967; p=0.033).

***Conclusion*:**

The resection of liver metastasis of colorectal adenocarcinoma leads to good survival rates. Lymphovascular invasion was the single prognostic factor related to the primary tumor. Synchronous disease and four or more metastasis were the most significant factors related to the secondary tumor.

## RESUMO

***Racional*:**

Cerca de metade dos pacientes com adenocarcinoma colorretal apresentará metástases hepáticas. Apesar da superioridade do tratamento cirúrgico, os pacientes com elas compõem um grupo muito heterogêneo.

***Objetivo*:**

Descrever o impacto de fatores relacionados ao tumor primário e ao secundário na sobrevida após ressecção de metástases hepáticas colorretais.

***Métodos*:**

Análise retrospectiva de base de dados mantida prospectivamente de pacientes operados.

***Resultados*:**

Foram realizadas 84 hepatectomias para ressecção de metástases hepáticas de adenocarcinoma colorretal em 73 pacientes no período. A sobrevida global e livre de doença em cinco anos foram de 48,8 e 27,5%, respectivamente. Os principais preditores de sobrevida foram grau de diferenciação (p=0,050) e invasão angiolinfática (p=0,021) do tumor primário, metástases sincrônicas (p=0,020), número (p=0,004), distribuição bilobar (p=0,019) e diâmetro máximo maior que 50 mm (p=0,027) dos nódulos hepáticos. Foram significativos a presença de invasão angiolinfática (HR=2,7; IC 95% 1,106-6,768; p=0,029), metástases sincrônicas (HR=2,8; IC 95% 1,069-7,365; p=0,036) e número de nódulos hepáticos igual ou superior a quatro (HR=1,7; IC 95% 1,046-2,967; p=0,033).

***Conclusão*:**

A ressecção de metástases hepáticas de adenocarcinoma colorretal proporciona melhora da sobrevida e os principais fatores prognósticos foram a invasão angiolinfática no tumor primário, metástases sincrônicas e quatro ou mais nódulos hepáticos.

## INTRODUCTION

It is expected that about 50% of the patients with colorectal adenocarcinoma will present with liver metastasis, 20% being diagnosed synchronous with the primary tumor^3^ ^,^ ^6^ ^,^ ^8^ ^,^ ^28^. Whenever feasible, liver resection presents with a 5-year overall survival between 24% and 64% in comparison with 10% to 11% of systemic chemotherapy alone^1^ ^,^ ^9^ ^,^ ^10^ ^,^ ^13^ ^,^ ^19^ ^,^ ^21^ ^,^ ^22^ ^,^ ^23^ ^,^ ^30^. Currently, patients with technically resectable nodules, a sufficient liver remnant, no or limited extra-hepatic disease and those considered fit to be submitted to major abdominal surgery are considered candidates for resection, though this accounts for only 20% of all metastatic patients^22^ ^,^ ^27^.

The main prognostic factors for overall survival after hepatic resection of colorectal metastasis are based on classical papers published between 1960 and 2000^9^ ^,^ ^10^ ^,^ ^13^ ^,^ ^18^ ^,^ ^21^. However, more recent publications failed to demonstrate adequate accuracy of these variables in current population^13^ ^,^ ^21^ ^,^ ^27^. Much of this disparity is attributed to widespread and improvement in chemotherapy, better patient selection and advancements in surgical techniques. Besides that, a great number of these prognostic factors are related to the hepatic disease and not to the primary tumor.

The aim of this study was to describe the overall survival in patients submitted to resection of colorectal liver metastasis (CRLM) and to describe predictive prognostic factors related to the primary and secondary tumors. 

## METHOD

A retrospective analysis of a prospectively maintained database of patients with CRLM submitted to resections with curative intent between January 2007 and August 2018 in the Hepatopancreatobiliary Unit at Hospital das Clínicas, Federal University of Minas Gerais, Belo Horizonte, MG, Brazil, a major public tertiary oncological center in Brazil. This study was approved by the institutional review board of the university (CAAE - 0913591260000).

The exclusion criteria were any histologic type other than adenocarcinoma, hepatic resection due to contiguous involvement of the liver by the primary tumor and patients submitted to surgery without curative intent.

The classification of the American Joint Committee on Cancer (AJCC, 8^th^ edition, 2017) was used for staging and the primary tumor were also described regarding tumor differentiation, perforation, lymphovascular and perineural invasion. Synchronous metastases were those detected simultaneously or within six months of the diagnosis of the primary tumor and the size was reported according to the pathology measurement^24^. The resection of four of more liver segments was considered a major hepatectomy. Postoperative complications were reported according to the Clavien-Dindo classification. 

### Statistical analysis

Categorical variables were described as frequencies and continuous variables with minimum and maximum, mean, median, standard deviation and interquartile range. The Chi-Square and Fisher exact test were used to test for homogeneity between variables. Survival analysis was performed with the Kaplan-Meier method and the log-rank test used to analyze differences between survival curves. To compare variables with survival a multivariate analysis was taken using those with a p=0,20 after univariate analysis and expressed as Hazard Ratio (HR). In all analysis a p value <0,05 was considered significant. Statistical analysis was performed using the IBM SPSS^®^ v23.0 (Chicago, IL, USA).

## RESULTS

In the given period, 73 patients were submitted to 84 liver resections of CRLM. Median follow-up was 44 months (3-140), with predominance of women (n=50; 68.5%), median age of 54 years (28-80) and without family history of colorectal cancer (n=60; 82.2%, Table 1). The 5-year disease-free and overall survivals were 27.5% and 48.8% respectively, with a median survival of 55 months. 

**TABLE 1:** Demographics and colorectal tumor factors in patients with CRLM submitted to liver resection with curative intent

Variable	n (%)	p *
Age · Median (min-max)	54 (28-80)	0.058
Gender · Female · Male	50 (68.5) 23 (31.5)	0.089
Primary tumor		
Location · Right-sided · Left-sided	19 (26.1) 54 (73.9)	0.111
T stage · T0 · T1 · T2 · T3 · T4 · Missing	1 (1.4) 1 (1.4) 8 (11.0) 44 (60.3) 16 (21.8) 3 (4.1)	0.214
N stage · N0 · N1 · N2 · NX · Missing	21 (28.8) 23 (31.5) 23 (31.5) 3 (4.1) 3 (4.1)	0.970
Tumor differentiation · Well/moderate · Low/signet ring cell · Missing	60 (82.2) 6 (8.2) 7 (9.6)	0.121
Lymphovascular invasion	34 (46.6)	0.078
Perineural invasion	26 (35.6)	0.042
CEA (ng/ml) · Median (min-max)	11 (0.2-489)	0.004
Liver metastasis		
CRLM classification · Synchronous · Metachronous	53 (72.6) 20 (27,4)	0.007
Number of nodules · 1 · 2-3 · = 4	43 (58.9) 17 (23.3) 13 (17.8)	0.012
Distribution · Unilobar · Bilobar	54 (74.0) 19 (26.0)	0.002
Diameter (mm) · Median (min-max)	40 (4 - 110)	0.066

*=Homogeneity between categories test; CRLM=colorectal liver metastasis; Min=minimum; Max=maximum; CEA=carcinoembryonic antigen; Ng/ml=nanogram/milliliter; mm=millimeter.

Regarding the primary tumor, most cancers were located on the left colon (n=54; 73.9%), with a T3 stage (n=44; 60.3%) and positive nodes (n=46; 63.0%). Well or moderately differentiated adenocarcinomas were the most frequent histology (n=60; 82.2%), with lymphovascular (n=34; 46.6%) and perineural invasions (n=36; 49.3%) present in almost half of the cases (Table 1).

Regarding the liver metastasis, most of them were synchronous (n=53; 72.6%), single nodules (n=43; 58.9%), with unilobar distribution (n=54; 74.0%) and a median size of 40 mm (1-110, Table 1).

The majority was submitted to neoadjuvant chemotherapy (n=65; 89.0%) and minor hepatectomies (n=38; 52.1%). In eight cases (11.0%) the colorectal and liver resections were performed simultaneously and in another seven (9.6%) the hepatic resection was done in a two-stage procedure (Table 2). The mean length of hospital stay was five days (3-30), with a reported complication rate of 24.7%, the majority being minor (17.8%) and with no post-operative mortality (Table 2). 

**TABLE 2:** Perioperative factors in patients with CRLM submitted to liver resection with curative intent

Variable	n (%)	p *
Neoadjuvant chemotherapy	65 (89.0)	0.058
Hepatectomy Minor · Segmentectomy (up to three) · Non-anatomical resections Major · Right hepatectomy · Left hepatectomy · Trisectionectomy · Non-anatomical resections · Segmentectomy (four or more)	40 (54.8) · 36 (49,3) · 4 (5,4) 33 (45.2) · 13 (17.8) · 6 (8.2) · 6 (8.2) · 6 (8.2) · 2 (2.7)	0.421
Simultaneous colorectal resection	8 (11.0)	0.686
Perioperative transfusion	7 (9.6)	0.683
Postoperative complications (Clavien-Dindo classification) [16] · 0 · I e II · III e IV · V	55 (75.3) 13 (17.8) 5 (6.8) 0 (0)	0.316
Length of stay (days) · Median (min-max)	5 (3 - 30)	0.565
Margin status · >1mm · >1mm	57 (78.1) 16 (21.9)	0.101

*=Homogeneity between categories test; CRLM=colorectal liver metastasis; Min=minimum; Max=maximum.

The most important prognostic factors related to survival after univariate analyses were: high tumor grade (p=0.050) and lymphovascular invasion (p=0.021) in the primary tumor and synchronous metastasis (p=0.020), four or more hepatic nodules (p=0.004), bilobar distribution (p=0.019) and a diameter of 50 mm or greater (p=0.027, Table 3).

**TABLE 3:** Univariate analysis of prognostic factors after CRLM resection with curative intent

Variable	OS (months) (P25; P75)	5-year OS (%)	p *
Location · Right-sided · Left-sided	41 (27-54) 61 (29-92)	40.1% 52.7%	0.282
T stage · T0-T2 · T3-T4	89 (60-119) 54 (44-63)	70.0% 44.7%	0.186
N stage · N0 · N+	88 (38-117) 50 (38-61)	64.0% 40.9%	0.228
Tumor differentiation · Well/moderately · Low/signet ring cell	55 (23-86) 39 (3-74)	49.6% 22.2%	0.050
Lymphovascular invasion · None · Present	77 (62-103) 48 (24-61)	71.7% 34.6%	0.021
Perineural invasion · None · Present	83 (44-121) 48 (35-60)	59.4% 37.5%	0.346
Simultaneous colorectal resection · No · Yes	55 (26-83) 30 (5-54)	49.5% 42.9%	0.208
CRLM classification · Synchronous · Metachronous	47 (38-55) 97 (70-123)	42.5% 64.6%	0.020
Number of nodules · 1 · 2-3 · = 4	83 (37-128) 79 (47-110) 33 (28-37)	58.3% 51.4% 17.3%	0.004
Distribution · Unilobar · Bilobar	83 (49-116) 47 (32-61)	61.3% 19.8%	0.019
Diameter (mm) · < 50mm · = 50mm	61 (31-90) 41 (25-56)	51.6% 42.4%	0.027
Neoadjuvant chemotherapy · No · Yes	68 (57-79) 55 (26-83)	64.3% 48.1%	0.480
Hepatectomy · Minor · Major	92 (28-115) 50 (27-72)	52.6% 45.4%	0.190
Perioperative transfusion · No · Yes	88 (26-126) 55 (27-82)	57.1% 48.5%	0.469
Postoperative complications (Clavien-Dindo classification) [16] · 0, I e II · III e IV	61 (31-90) 39 (19-58)	50.0% 40.0%	0.300
Margin status · > 1mm · =< 1mm	79 (46-111) 44 (33-54)	54.2% 28.9%	0.164

*=log-rank test; CRLM=colorectal liver metastasis; OS=overall survival; P=percentile; mm=millimeter

After multivariate analysis, the only factor related to the primary tumor that remained statistically significant for worst prognosis was presence of lymphovascular invasion, with a HR of 2.7 (CI 95% 1.106-6.768; p=0.029). Regarding the secondary tumor, synchronous metastasis and four or more nodules were also significant (Table 4).

**TABLE 4:** Multivariate analysis of prognostic factors after CRLM resection with curative intent

Variable	HR	CI 95%	p
Tumor differentiation · Well/moderately · Low/signet ring cell	- 2.9	0.840 - 10.437	0.091
Lymphovascular invasion · None · Present	- 2.7	1.106 - 6.768	0.029
CRLM classification · Synchronous · Metachronous	2.8 -	1.069 - 7.365	0.036
Number of nodules · < 4 · = 4	- 1.7	1.046 - 2.967	0.033
Diameter >= 50mm	-	-	0.361
Hepatectomy · Minor · Major	-	-	0.443
Margin status · > 1mm · =< 1mm	-	-	0.230

CRLM=colorectal liver metastasis; HR=hazard ratio; CI=confidence interval; mm=millimeter

## DISCUSSION

The 5-year overall survival rate following liver resection for CRLM has improved from 24% to up to 64% over time^1^ ^,^ ^9^ ^,^ ^10^ ^,^ ^13^ ^,^ ^19^ ^,^ ^21^ ^,^ ^22^ ^,^ ^23^ ^,^ ^30^. The surgical treatment of CRLM in the present study presents with a 5-year overall survival of 48.8% and the most important prognostic factors after multivariate analysis were lymphovascular invasion in the primary tumor, CRLM diagnosed within six months of the colorectal cancer and the presence of four or more nodules. 

Colorectal tumor location has been pointed out as an important prognosis factor for both localized and metastatic patients^4^ mainly because of singularities in pathological and molecular phenotypes and, consequently, different chemotherapy treatment response. In this study, the survival difference between right and left colon cancer (5-year overall survival of 40.1% and 52.7%, respectively) did not prove to be statistically significant after univariate analysis (p=0.282). Although a retrospective cohort of 221 patients in 2018 corroborate this finding, two recent meta-analysis showed a worse prognosis of right sided colon cancer with a HR of up to 1.39 (CI 95% 1.28-1.51; p<0,001)^12^ ^,^ ^15^ ^,^ ^25^ ^,^ ^29^ ^,^ ^31^. On the other hand, Yamashita et al. ^30^ demonstrated that KRAS gene mutational status was an independent survival prognostic factor regardless of primary tumor location in patients submitted to resections of CRLM.

The T and N stages are also frequently considered prognostic factors of colorectal adenocarcinoma. The nodal stage is present on the three classical prognostic scores for CRLM treatment (Nordlinger, Fong and Basingstoke index), but a paper by Reissfelder et al. ^22^ proposed to validate these factors in a current patient cohort and failed to demonstrate its reproducibility^7^ ^,^ ^9^ ^,^ ^18^ ^,^ ^21^. The lymph node stage is a highly variable factor that can be influenced by a number of features such as patient age, immune response, primary site and, specially, neoadjuvant therapy, much more commonly used nowadays^7^. Therefore, in resemblance to the primary tumor location, it is unclear whether nodal status has a significant relation with survival after CRLM resections. In fact, these two variables were not associated with survival in our study.

Lymphovascular invasion in the colon or rectum, however was the single most significant prognostic factor related to the primary tumor after both univariate and multivariate analysis, with a HR of 2.7 (CI 95% 1.106-6.766; p=0.029). This pathological finding is usually related to tumor grading, local invasion and local recurrence, though not frequently pointed out as an important factor related to the primary tumor in metastatic setting^2^. A retrospective study performed in 2012 corroborates these data showing a survival of 48 months in patients with lymphovascular invasion, compared to 69 months for those without it (p<0,0001)^5^.

Regarding the metastatic disease, the occurrence of liver nodules within six months of diagnosis of the primary tumor was also an independent worse prognosis factor of survival. There is no consensus on the literature on the exact definition of a synchronous metastasis. It has been defined from only those diagnosed simultaneously with the primary as well as up to 30 months afterwards^9^ ^,^ ^10^ ^,^ ^13^ ^,^ ^24^ ^,^ ^28^. Because of these conceptual disparities, the impact of this factor on survival is also a matter of debate, and a few works have been unable to demonstrate its significance when using a cutoff of up to three months^17^ ^,^ ^22^. Despite this fact, synchronous metastasis either represent a delayed diagnosis or, more commonly, a more aggressive neoplasia with a worse tumor biology, hence, poorer prognosis. On the other hand, it is expected that metachronous metastasis are diagnosed earlier and possibly in small number and size.

The association of size, number and bilobar distribution of CRLM with survival is also straightforward and was significant after univariate analysis, even though only the number of four or more nodules have remained significant after multivariate analysis. In a resemblance of the above mentioned discussion on the synchronous definition, there is great variability on the cutoffs values of these variables^10^ ^,^ ^11^ ^,^ ^13^ ^,^ ^19^ ^,^ ^21^ ^,^ ^22^. Interestingly, the widespread indication of neoadjuvant therapy may have impacted the relevance of these factors on survival, since it is not uncommon for this patients to present with a shrinkage of the nodules and even its disappearance in follow-up imaging. A retrospective analysis by John et al.^11^ including 432 patients with an incidence of neoadjuvant chemotherapy of 60% has failed to demonstrate the impact of number and size on survival. In this case series 89% of patients has been submitted to neoadjuvant chemotherapy, a much higher number when compared to classical series that ranges from 0 to 60% of the cohort^17^ ^,^ ^21^ ^,^ ^30^.

Surgical resection margins under 1 mm were found in 21.9% of resections, but did not impacted on survival. The cut-off value of 1 mm for surgical margins is also a matter of debate in the literature, with an incidence of up to 37.8% of nodules being within this distance from liver transection^14^. The pathology assessment of resection margins can be highly compromised after liver resection due to carbonization and loss of the surrounding parenchyma, as well as fracture of the liver tissue during processing of the material, conceivably leading to a higher rate of positive margins^19^. However, a 2017 meta-analysis that enrolled 11147 patients regarding this matter demonstrated an improvement in 5- and 10-year overall survival rates when margins were >1 cm distance from surgical transection line (RR=0.91, p=0.003; RR=0.94, p=0.054, respectively)^14^. On the other hand, a series of works have questioned this impact when considering the relation of submillimeter resection margin with KRAS mutational status, adjuvant chemotherapy treatment and the size of the tumor assessed^13^ ^,^ ^16^ ^,^ ^26^.

The main limitations of this study can be attributed to its retrospective nature and the small sample size. The adjuvant chemotherapy regimens were also not assessed, possibly leading to different outcomes, as well as adjuvant chemotherapy for the primary tumor. Likewise, imaging follow-up of clinical response after neoadjuvant chemotherapy was not assessed in this study. Prospective studies with a greater number of patients are needed to better draw consensus on these prognostic factors, especially regarding the primary tumor.

## CONCLUSION

The resection of liver metastases of colorectal adenocarcinoma leads to high survival rates of up to 64%. A great number of factors are used in survival prediction, especially those related to the metastatic disease, but the assessment of lymphovascular invasion in the primary tumor is a widely available and easily assessed variable that significantly impacts survival.
